# Disparities in Cutaneous Melanoma Diagnosis and Survival Among American Indian and Alaskan Native Patients: A Systematic Review and Meta‐Analysis

**DOI:** 10.1002/jso.70136

**Published:** 2025-11-24

**Authors:** Rena A. Li, Antoinette T. Nguyen, Kethan Bajaj, Brigid M. Coles, Robert D. Galiano

**Affiliations:** ^1^ Northwestern University Feinberg School of Medicine Chicago Illinois USA; ^2^ University of Rochester School of Medicine and Dentistry Rochester New York USA

**Keywords:** American Indian and Alaska Native, disparities outcomes, health disparities, melanoma, meta‐analysis, Native Americans, skin cancer

## Abstract

**Backgrounds and Methods:**

American Indian and Alaska Native (AI/AN) populations face significant health disparities across multiple cancer types, yet melanoma‐specific outcomes remain under‐investigated. A comprehensive search of Embase, Scopus, and PubMed identified 20 studies meeting inclusion criteria. Three meta‐analyses were conducted using random effects models to assess: (1) adjusted hazard ratios for mortality risk, (2) adjusted odds ratios for late‐stage diagnosis, and (3) age‐adjusted incidence rates.

**Results:**

The meta‐analysis revealed significant disparities in melanoma outcomes for AI/AN patients. AI/AN patients demonstrated a 43% higher mortality risk compared to white patients (pooled aHR = 1.43, 95% CI: 1.12–1.82, *p* = 0.0041) and a 75% higher likelihood of late‐stage diagnosis (pooled adjusted OR = 1.75, 95% CI: 1.16–2.65, *p* = 0.0080). AI/AN patients consistently presented with worse prognostic factors including higher Breslow thickness, increased ulceration rates, and more advanced disease stages.

**Conclusion:**

This study provides the first meta‐analytic evidence demonstrating statistically significant disparities in melanoma outcomes among AI/AN populations. Systemic barriers include insurance disparities, geographic isolation, treatment delays, and limited access to specialized dermatologic care.

**Discussion:**

These findings justify targeted interventions including enhanced screening programs, improved healthcare infrastructure, and policy reforms to address insurance and access barriers affecting AI/AN communities.

## 1 Introduction

Melanoma is a serious and potentially fatal form of skin cancer that arises from melanocytes, the pigment‐producing cells in the skin [1]. While early‐stage melanomas are highly treatable, survival drops significantly with disease progression. The 5‐year survival rate for patients diagnosed with melanoma‐in‐situ is 97%, compared to only 30% for those diagnosed at stage IV [2]. In the US, prompt treatment following diagnosis is also associated with improved outcomes [3]. Disparities in healthcare access and utilization may prevent early diagnosis in certain populations, leading to poorer prognosis.

American Indian and Alaska Native (AI/AN) populations face structural barriers, such as geographic isolation, underfunded healthcare, poverty, and institutional mistrust, which contribute to delayed diagnosis and worse outcomes [4, 5, 6, 7]. Despite broader attention to cancer disparities, melanoma outcomes in AI/AN communities remain under‐studied, often due to limited statistical power in smaller populations.

This systematic review and meta‐analysis seek to address this gap by analyzing and synthesizing existing quantitative evidence related to melanoma outcomes in AI/AN populations. We focus on disparities in stage at diagnosis and survival rates, as these metrics serve as key indicators of access to and quality of care. While we recognize the barriers leading to these outcomes are complex and multifactorial, quantifying these inequities is an essential first step in addressing them. This review aims to inform future research, healthcare policy, and clinical efforts directed at improving early detection and reducing melanoma‐related mortality among AI/AN individuals.

## 2 Methods

This systematic literature review was conducted under the Preferred Reporting Items for Systematic Reviews and Meta‐Analyses (PRISMA) guidelines and was registered in the PROSPERO database (ID: CRD420251015326). This study aimed to identify and analyze studies addressing disparities in cutaneous melanoma survival rates and diagnosis among AI/AN patients. We applied a comprehensive search strategy across Embase, Scopus, and PubMed. The search included articles published starting in 2000 up to the present time. The search terms combined both MeSH terms and free‐text keywords related to cutaneous melanoma and American Indian/Alaska Native populations.

**Figure 1:**
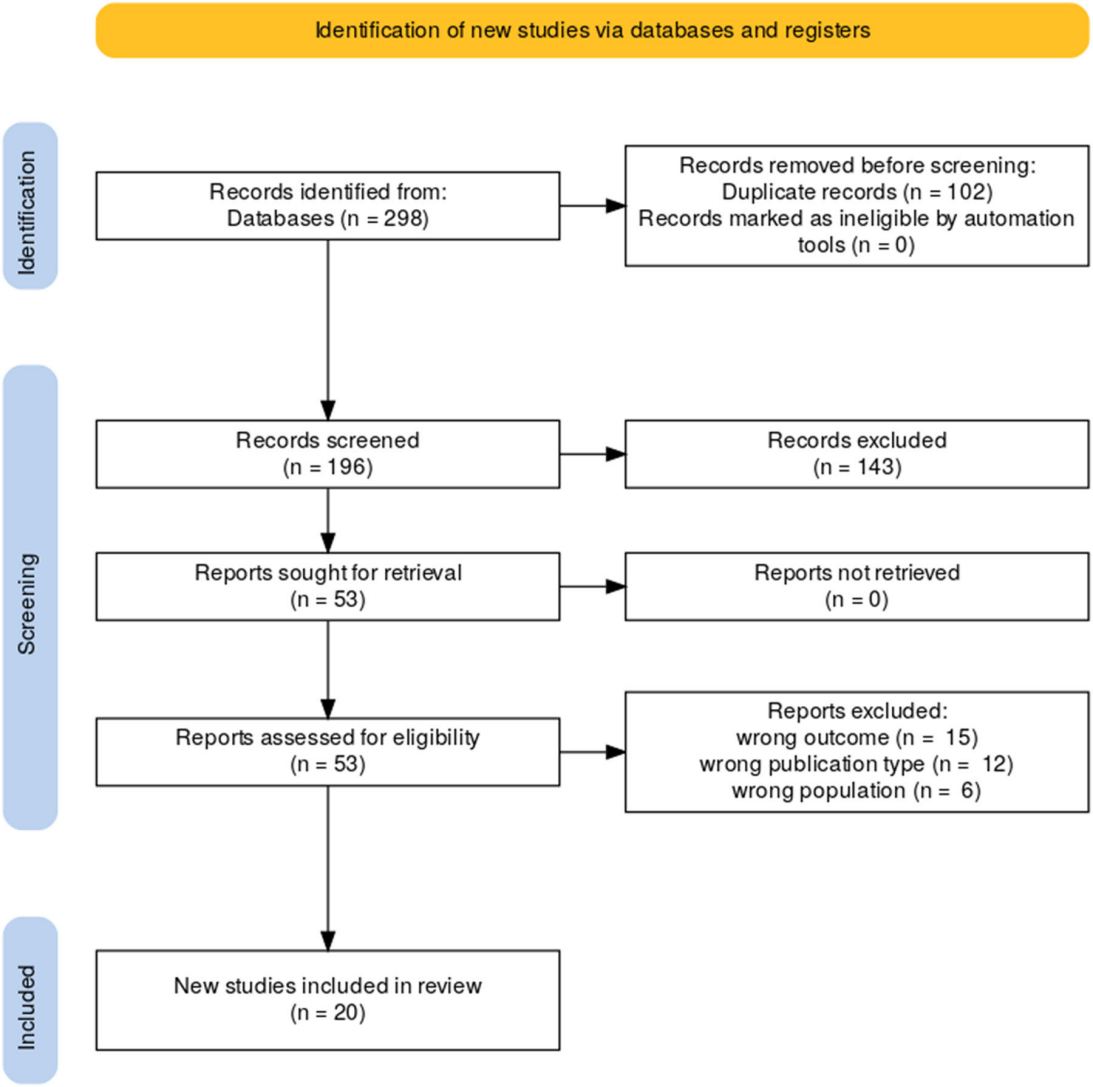
PRISMA Diagram.

A systematic search across Embase, Scopus, and PubMed identified 298 records, of which 196 unique studies were screened. After title/abstract screening and full‐text review, 20 studies were included (Figure 1). Two authors conducted the initial screening, with a third resolving conflicts. Studies were included in the review if they met the following criteria: they focused on cutaneous melanoma, included AI/AN patients in the study population, and assessed disparities in melanoma prognostic factors, survival rates, or related outcomes. Data extraction captured details such as author, year of publication, study design, population characteristics (sample size, age, etc), and key findings on melanoma such as thickness, stage at diagnosis, ulceration, and mortality risk.

A risk‐of‐bias assessment was conducted for each study (Table 1). Two reviewers independently evaluated selection, confounding, outcome measurement, data completeness, and overall biases across studies using the Appraisal tool for Cross‐sectional studies (AXIS) or the Newcastle‐Ottawa Scale (NOS) for cohort studies. Differences in assessments were addressed through a third reviewer to limit bias while evaluating the study quality.

**Table 1:** Risk‐of‐bias Assessment.

Study	Study design	ROB tool	Selection bias	Confounding	Outcome measurement	Data completeness	Overall risk
Cormier et al. 2006	Retrospective cross‐sectional study of SEER database	AXIS	Low	Moderate	Low	Moderate	Moderate
Weir et al. 2011	Retrospective cross‐sectional study of NPCR	AXIS	Low	Low	Low	Moderate	Low
Wu et al. 2011	Retrospective cross‐sectional study of population‐ based cancer registries	AXIS	Low	Moderate	Low	Moderate	Moderate
Martinez et al. 2012	Retrospective cross‐sectional study of SEER database	AXIS	Moderate	High	Low	Moderate	High
Bristow et al. 2013	Retrospective cross‐sectional database study	AXIS	Low	Low	Moderate	Low	Low
Campbell et al. 2014	Retrospective cross‐sectional study of the OCCR	AXIS	Low	Moderate	Low	Moderate	Moderate
Baldwin et al. 2016	Retrospective cross‐sectional study of the OCCR	AXIS	Low	Moderate	Low	Moderate	Moderate
Qian et al. 2021	Retrospective cross‐sectional study of SEER database	AXIS	Low	Moderate	Low	Low	Low
Melkonian et al. 2022	Retrospective cross‐sectional study of CDC and SEER databases	AXIS	Low	Moderate	Low	Moderate	Moderate
Rosenthal et al. 2022	Retrospective cohort study of California Cancer Registry	NOS	Low	Moderate	Low	Moderate	Moderate
Fernandez et al. 2023a	Retrospective cohort study of the NCDB	NOS	Moderate	Moderate	Moderate	Moderate	Moderate
Fernandez et al. 2023b	Retrospective cohort study of the NCDB	NOS	Moderate	Moderate	Moderate	Moderate	Moderate
Joshi et al. 2023	Retrospective cohort study of SEER database	AXIS	Low	Moderate	Low	Moderate	Moderate
Popp et al. 2024	Retrospective cross‐sectional study of the NCDB	AXIS	Moderate	Moderate	Low	Moderate	Low
Zhang et al. 2024	Retrospective cross‐sectional study of the SEER database	AXIS	Low	Moderate	Moderate	Moderate	Moderate
Townsend et al. 2024	Retrospective cross‐sectional study of the NPCR and SEER databases	AXIS	Low	Moderate	Low	Moderate	Moderate
Fernandez et al. 2024	Retrospective cohort study of the NCDB	NOS	Moderate	Moderate	Moderate	Moderate	Moderate
Taparra et al. 2024	Retrospective cohort study of the NCDB	NOS	Moderate	Low	Low	Moderate	Moderate
Taylor et al. 2025	Retrospective cross‐ sectional study of the SEER Database	AXIS	Low	Moderate	Low	Moderate	Moderate
Kim et al. 2025	Retrospective cross‐sectional study of the SEER database	AXIS	Low	Moderate	Moderate	Low	Moderate

## 3 Meta‐Analysis

Three meta‐analyses were conducted to quantitatively aggregate important statistical findings across studies that were sufficiently similar in population characteristics, study design, and reported melanoma‐related outcomes. The first assessed adjusted hazard ratios (aHRs) and 95% confidence intervals (CIs) extracted from studies that compared the mortality risk of AI/ANs with cutaneous melanoma compared to white patients. For this meta‐analysis, seven studies were selected: Cormier et al. (2006), Qian et al. (2021), Fernandez et al. (2023), Fernandez et al. (2023), Joshi et al. (2023), Taparra et al. (2024), and Taylor et al. (2025) [8, 9, 10, 11, 12, 13, 14].

The second meta‐analysis assessed adjusted odds ratios (OR) for late‐stage diagnosis reported in three studies: Cormier et al. (2006), Joshi et al. (2023), and Taparra et al. (2024) [8, 12, 13]. Joshi et al. and Taparra et al. examined the odds of diagnosis at stages 3 or 4, while Cormier et al. reported on stage 4 diagnoses among AI/ANs.

The last meta‐analysis was performed on reported annual age‐adjusted incidence rates per 100,000 across six studies: Cormier et al. (2006), Weir et al. (2011), Wu et al. (2011), Campbell et al. (2014), Townsend et al. (2024), and Taylor et al. (2025) [8, 14, 15, 16, 17, 18]. Incidence rates and 95% CIs were extracted and aggregated in the meta‐analysis.

All meta‐analyses were performed using the random effects model under the “meta” package in R to account for the heterogeneity across the included studies. Forest plots were created to illustrate the overall effect estimate and evaluate heterogeneity among the studies. Funnel plots demonstrate the presence of potential publication bias.

## 4 Results

This systematic review and meta‐analysis included 21 studies to investigate disparities in melanoma outcomes among AI/AN patients compared to white patients. Our analysis has been divided into three major parts: higher mortality risk, worse prognostic indicators, and disparities in access to healthcare. The main findings of these studies have been aggregated in Table 2.

**Table 2:** Summary of included studies.

Study	Methods	Key findings	Conclusions	Limitations
Cormier et al., 2006	Data from the SEER program from 1992 to 2002 were extracted for patients diagnosed with primary cutaneous invasive melanoma. Race/ethnicity was defined as white, Hispanic, African American, American Indian, and Asian/Pacific Islander.	American Indians had the second‐highest annual age‐adjusted melanoma incidence rates among minorities at 1.6 (95% CI: 1.2–2.1) per 100,000 persons. AI/AN patients were also diagnosed at a younger age relative to other races at 52 years (*p* < 0.001) and had a higher median Breslow thickness than white patients at 0.77 mm versus 0.66 mm. 15.4% of AI/AN patients presented at stage IV compared to only 3.9% of whites (*p* < 0.001). AI/AN patients had an odds ratio of 3.38 for presenting at stage IV relative to whites. AI/AN patients also had a lower 5‐year overall survival rate (*p* < 0.001) and melanoma‐specific survival rate of 69.8% and 81.0%, respectively, relative to 79.3% and 89.6% for whites. AI/AN patients had a 2.60‐fold greater risk of melanoma‐specific mortality compared to whites (*p* < 0.05), but this difference became insignificant after adjusting for additional factors such as marital status, year of diagnosis, etc.	Despite lower incidence rates of melanoma in minority groups, these patient populations consistently face poorer overall and disease‐specific survival compared with white patients. These worsened outcomes can be attributed to the higher likelihood of late‐stage diagnosis and differences in frequency of histology type across racial groups.	The SEER program does not have access to important factors that can affect one's outcomes, such as socioeconomic data, comorbidities, and access to healthcare.
Weir et al., 2011	Data from cancer registries across both SEER and NPCR from 1999‐2006 were extracted for melanoma patients aged 15–39 years. Race/ethnicity was defined as Non‐Hispanic white, Hispanic white, black, American Indian/Alaskan Natives, and Asian Pacific Islanders. Unspecified race categories were combined.	AI/AN patients had the highest age‐adjusted incidence rates of 2.22 per 100,000 males and 2.89 per 100,000 females across all minority groups. Remaining site and histology analyses were conducted by combining nonwhite patient data (black, AI/AN, API). Incidence rates were higher for females across all racial groups.	In younger populations, AI/AN patients may be more susceptible to developing melanoma relative to patients of other minority groups.	The number of AI/AN patients in this study was too low to be analyzed as a standalone group, leading to the combination of multiple nonwhite racial groups.
Wu et al., 2011	Data from cancer registries across both SEER and NPCR from 1999 to 2006 were extracted for melanoma patients. Race/ethnicity was defined as white, Hispanic, black, AI/AN, and API. Data with no specified race were excluded from the race‐specific analysis.	AI/AN patients had the second highest age‐adjusted incidence rates for melanoma of 4.52 per 100,000 across all minority groups, second to Hispanics with an incidence rate of 4.68 per 100,000. AI/AN patients were younger at diagnosis than white and black patients, with a median age at diagnosis of 54 years. AI/AN patients were more likely to be diagnosed at later stages and have a higher Breslow thickness than white patients. 7.7% of AI/AN patients were diagnosed at the distant stage, relative to 3.7% of whites. 62.5% of whites and 48.1% of AI/AN patients had a thickness of less than or equal to 1.0 mm. Conversely, 4.6% of whites had a thickness of greater than 4.0 mm, while 11.2% of AI/AN patients had the same thickness. AI/AN had an 84.9% 5‐year melanoma‐specific survival rate compared to 90.0% for whites. The majority of survival rates by histology, site, and thickness were unable to be calculated for the AI/AN group.	This study reiterates the disparity in stage at diagnosis and Breslow thickness for minority patients. Nonwhite patients consistently had lower survival rates relative to their white counterparts.	Small sample sizes for the AI/AN group led to limited in‐depth analyses. Additionally, the databases used in this study are subject to potential underreporting of melanoma and misclassification of race. Lastly, the majority of cases did not have a reported histology, limiting analysis of this variable.
Martinez et al., 2012	Data from the SEER program from 2004 to 2008 were extracted for patients who underwent surgery for cutaneous melanoma with a Breslow thickness of 1 to 4 mm. Race/ethnicity was defined as white, black, Asian Hispanic, Native American, and unknown.	A multivariate logistic regression showed that Native American patients had the lowest likelihood of undergoing sentinel lymph node biopsy (OR = 0.19, *p* = 0.019) across all racial groups analyzed. More general geographic trends showed that patients in the south were 46% less likely than those in the western US to receive an SLNB.	Patients with a positive SLNB are typically offered a lymphadenectomy, which has been shown to improve melanoma‐specific survival. The decreased likelihood for AI/AN patients to undergo SLNB could play a role in the lowered rates of melanoma‐specific survival reported in other studies.	The SEER program does not have access to important factors that can affect one's outcomes, such as socioeconomic data, comorbidities, and access to healthcare.
Bristow et al., 2013	Publicly available multiple‐cause‐of‐death data were extracted from US death certificates from 1990 to 2008. People with “malignant melanoma of the skin” as a cause of death were included in the analysis. Race/ethnicity was defined by Non‐Hispanic white, black, Hispanic, Asian/Pacific Islander, and Native American/Alaskan Native.	AI/AN patients had the highest age‐adjusted mortality rate of 0.88 per 100,000 among all minority groups. The difference in mortality rates was significant between both AI/AN and blacks and AI/AN and API patients. The average age at death was 62.5 years and 66.1 years for AI/AN patients and white patients, respectively.	AI/AN patients had elevated age‐adjusted mortality rates and were more likely to die at a younger age relative to white, black, and API patients. The mortality rate relative to the incidence rates among the minority groups included in this study suggests that these patients may be at a higher risk for melanoma‐related death.	Death certificates can be affected by errors, incompleteness, or the subjective nature of the reporting done by physicians or coroners. Additional factors such as medical history or other important social circumstances are not reported. Lastly, the mortality rates calculated in this study could be affected by inaccurate estimates of populations.
Campbell et al., 2014	Data from 1997–2001 to 2005–2009 were extracted from the Oklahoma Central Cancer Registry. Only American Indian/Alaska Natives and white patients were included in this study.	Age‐adjusted melanoma incidence rates were 27.4 and 16.4 per 100,000 for whites and AI/AN patients, respectively. From 1997‐2001 and 2005‐2009, AI/AN patients consistently were diagnosed at later stages than white patients (*p* = 0.030). However, both groups had favorable trajectories between these two periods. From 1997‐2001, 20.6% of whites and 27.5% of AI/AN patients had late‐stage diagnoses, compared to 14.0% and 20.0% for whites and AI/AN in 2005–2009, respectively. Across all cancer types, the study found that 40.7% of AI/AN patients included in the study had Medicare, with private insurance being the second most common primary payer (24.7%). More AI/AN patients relied on Medicaid than white patients (7.8% vs. 4.1%).	This study demonstrated disparities in multiple cancer types between AI/AN and white patients, which may in part be attributed to differences in insurance coverage and proximity to healthcare resources. For melanoma specifically, AI/AN patients were statistically more commonly diagnosed at a later stage, which also likely plays a role in worsened outcomes relative to white patients.	A large percentage of cases were missing stage data. This study did not include mortality rates of any cancer type. Additionally, since many AI/AN people live in rural areas, the observed disparities could be a result of differences in geographic location.
Baldwin et al., 2016	Data from 2000 to 2008 were extracted from the Oklahoma Central Cancer Registry. Race/ethnicity was defined as White Non‐Hispanic, White Hispanic, African American, AI/AN, and Asian/Pacific Islander.	20.1% of AI/AN patients had late‐stage diagnoses of melanoma compared to 15.1% of white non‐Hispanics (*p* = 0.02). AI/AN patients also had the highest period prevalence among all minority groups of 88.0 (95% CI: 85.1–91.4) per 100,000. Both the crude mortality rate (*p* = 0.0003) and age‐adjusted mortality rate for AI/AN patients in Oklahoma were significantly higher than the national average. AI/AN patients in Oklahoma also had a higher crude mortality rate (1.2) for melanoma than white Hispanics (0.4, *p* = 0.0053) and African Americans (0.3, *p* < 0.0001). AI/AN patients had an age‐adjusted mortality rate of 1.7 in Oklahoma and 1.0 in the United States, both of these values being the highest among all minority groups measured in this study.	This study reinforces that AI/AN patients are diagnosed at later stages of melanoma compared to white non‐Hispanics. The analysis showed that Oklahomans may be diagnosed at later stages, since the crude and age‐adjusted mortality rates in Oklahoma were either equal to or greater than the national rates. The mortality rate for AI/AN patients was 2–3 times greater than other minority groups, indicating not only a disparity between AI/AN and whites but also among other nonwhite groups.	Mean survival was used as an indicator of mean duration for prevalence calculations, which could skew the results. Additionally, the OCCR has previously been shown to overestimate survival data. Lastly, the small values for several races can limit the comparisons between groups.
Qian et al., 2021	Data from 1975 to 2016 were extracted from the SEER database for cutaneous, mucosal, and uveal melanoma patients. Race/ethnicity was defined as Hispanic, Non‐Hispanic white, Non‐Hispanic black, Non‐Hispanic Asian or Pacific Islander, and Non‐Hispanic American Indian/Alaska Native.	18.6% of NHAIANs were diagnosed at regional or distant stages compared to only 12.6% of Non‐Hispanic whites. Melanoma‐specific survival improved from the 1975 to 2000 period to the 2010–2016 period for both NHAIANs (84.9% to 86.0%, *p* = 0.20) and NHWs (88.1% to 92.9%, *p* < 0.001). Although NHAIANs had a positive trend in survival, the difference compared to whites was still significantly worse (*p* < 0.05). NHAIANs also had greater skin thickness (1.49 mm) and a higher proportion of skin ulceration (16.2%) relative to white patients (1.22 mm, 12.8%). White patients had the highest median follow‐up period of 86 months, with NHAIANs having a year less of follow‐up at only 74 months. Hazard ratios adjusted for age, gender, primary site, histologic subtype, and stage showed that NHAIANs were at increased risk relative to whites. From 1975 to 2000, 2000–2009, and 2010–2016, the aHRs for NHAIANs were 1.12 (95% CI: 0.78–1.60), 1.15 (95% CI: 0.85–1.55), and 1.61 (95% CI: 1.12–2.32), respectively. Risk for NHAIANs was particularly high at the localized (2.14, 95% CI: 1.02–4.50) and regional stages (2.86, 95% CI: 1.66–4.94) between 2010 and 2016.	Despite modest improvements in melanoma‐specific survival for AI/AN patients, disparities in survival rates, skin thickness, ulceration, and follow‐up persist relative to white patients. AI/ANs are at higher risk of melanoma‐specific mortality relative to whites in the most recent analyzed time period, specifically when diagnosed at the localized or regional stage.	The SEER program does not include information on immunotherapy, which is an important factor in melanoma treatment that could not be analyzed. Socioeconomic factors such as income or insurance coverage were also not accessible through the SEER database.
Melkonian et al., 2022	Data from 1999 to 2017 were extracted from the National Program of Cancer Registries of the CDC and SEER databases. Only Non‐Hispanic AI/AN and Non‐Hispanic white patients were considered.	The age‐adjusted melanoma incidence rate was 9.1 per 100,000 for AI/AN patients and 30.6 per 100,000 for Non‐Hispanic whites from 1999 to 2017. During this period, the incidence rate increased by 53% among white females and 186% among AI/AN females, and by 54% among white males and 98% among AI/AN males.	The increasing incidence rates for AI/AN patients, particularly for females, could indicate an expanding racial disparity between urban AI/AN and white populations.	The databases used in this study are subject to racial misclassifications. Additionally, there may be a lack of AI/AN representation for those who live outside of the counties included in the cancer registries. Lastly, socioeconomic factors such as income or insurance coverage were not accessible through the SEER and NPCR databases.
Rosenthal et al., 2023	Data from 2009 to 2014 were extracted from the California Cancer Registry for adults diagnosed with stage I‐IV melanoma. Follow‐up was performed through 2017. Race/ethnicity were defined as Non‐Hispanic White, Hispanic, African American/Black, Native American, and other.	Due to the small sample size of Native Americans (*n* = 27), no meaningful statistical comparisons could be made. The melanoma‐specific mortality rate for Native Americans with OPI was reported as 22.7 deaths per 1000 person‐years (95% CI: 0.6–126.7). Hazard ratios adjusted for age, sex, race/ethnicity, SES, county of residence, years of diagnosis, health insurance payer, stage at diagnosis, and treatment (primary surgical treatment, chemotherapy, radiation therapy, and hormone therapy) were calculated. The adjusted hazard ratio for melanoma‐specific mortality for Native Americans with private insurance outside of Kaiser was 1.84 (95% CI: 0.26–13.25). The aHR for all Native Americans was 0.98 (95% CI: 0.14–7.00).	Within an insured population, race/ethnicity was not associated with melanoma‐specific mortality, suggesting that previously reported racial disparities may be largely driven by lack of insurance. SES, however, was a significant determinant of mortality. Integrated systems like KPSC may mitigate SES‐related disparities in melanoma outcomes.	The database lacked information on comorbidities, health behaviors (e.g., smoking, physical activity), and post‐diagnosis care location. Treatment details were limited, and small sample sizes for Native Americans precluded meaningful subgroup analyses. The reliance on registry‐defined race/ethnicity may also introduce misclassification bias.
Fernandez et al., 2023a	Data from cutaneous melanoma cases diagnosed in females from 2004 to 2018 were extracted from the National Cancer Database. Race/ethnicity was defined as Non‐Hispanic White, Non‐Hispanic Black, Non‐Hispanic Asian, Non‐Hispanic American Indian/Alaska Native, and Hispanic.	Hazard ratios were adjusted for age, race/ethnicity, primary site, insurance status, Charlson‐Deyo comorbidity score, stage at diagnosis, melanoma histologic subtype, ulceration, surgery, systemic therapy, and chemotherapy. Non‐Hispanic AI/AN women were found to have a slightly decreased mortality risk (aHR = 0.95, *p* = 0.823) compared to white women as the reference. Factors such as ulceration (HR: 1.77, *p* < 0.001), stage II (HR: 2.13, *p* < 0.001), III (HR: 3.49, *p* < 0.001), or IV (HR: 10.26, *p* < 0.001) at diagnosis, and insurance status increased one's risk of melanoma‐related mortality.	There were limited AI/AN‐specific findings from this study. The primary difference between AI/AN and white patients was not statistically significant.	This study does not include melanoma‐specific survival rates. It is also impacted by changes in staging criteria throughout the observed period and small sample sizes for nonwhite groups.
Fernandez et al., 2023b	Data from cutaneous melanoma cases diagnosed in males from 2004 to 2018 were extracted from the National Cancer Database. Race/ethnicity was defined as non‐Hispanic White, non‐Hispanic Black, non‐Hispanic Asian, non‐Hispanic American Indian/Alaska Native, and Hispanic.	AI/AN males had the lowest median age at diagnosis of 57 years across all racial groups (*p* < 0.001). They also faced the highest median distance to the diagnosing institution (*p* < 0.001). A significant portion of AI/ANs had Medicare (43.4%) or private insurance (43.4%) as their primary payer. 7.2% of AI/ANs were covered by Medicaid, compared to only 2.6% for white patients, and 6.1% of AI/ANs did not have insurance. They had lower median incomes than other racial groups, including Hispanic, Asian, and white patients, with only 23% of AI/ANs earning more than $63,000 a year. The median measured Breslow thickness was 1.15 mm (IQR: 0.42–3.06) for AI/ANs and 0.88 mm (IQR: 0.40–2.00) for whites. 29.1% of AI/ANs were diagnosed at stage III or IV, as opposed to 21.1% for white patients. More AI/AN patients had a positive lymph node status (19.4%) and skin ulceration (24.7%) than white patients (13.8% positive lymph node status and 20.6% skin ulceration). Hazard ratios were adjusted for age, race, ethnicity, primary site, insurance status, Charlson‐Deyo comorbidity score, stage at diagnosis, melanoma histology, ulceration, surgery, systemic therapy, and radiation. AI/AN males had an aHR of 1.11 with whites as the reference group (*p* = 0.541). Factors such as ulceration (HR: 1.61, *p* < 0.001), stage II (HR: 1.9, *p* < 0.001), III (HR: 3.17, *p* < 0.001), or IV (HR: 9.09, *p* < 0.001) at diagnosis, and insurance status increased one's risk of melanoma‐related mortality. 5‐year overall survival rates were 75.1% for white males and 68.5% for AI/AN males.	AI/AN males were diagnosed at a younger age and with more advanced disease compared to white males, with higher Breslow thickness, later stage, and greater rates of ulceration and lymph node involvement. Despite similar treatment patterns, they had lower 5‐year overall survival. While the adjusted hazard ratio was not statistically significant, disparities in stage at diagnosis and socioeconomic factors may contribute to worse outcomes.	This study does not include melanoma‐specific survival rates. It is also impacted by missing data and small sample sizes for nonwhite groups.
Joshi et al., 2023	Data from 2000 to 2019 were extracted from the SEER database for cutaneous melanoma patients. Race/ethnicity was defined as Hispanic, white, black, Asian or Pacific Islander, and American Indian/Alaska Native.	A larger proportion of AI/AN patients were diagnosed at stage III or IV (16.5%) compared to their white counterparts (10.4%), but this difference was not statistically significant. In a univariate logistic regression analyzing late‐stage diagnosis, AI/AN patients had an odds ratio of 1.71 (*p* = 0.033) with white patients as the reference. When using a multivariate analysis, this difference increased and approached significance (OR = 1.76, *p* = 0.060). AI/ANs faced the highest risk of lower overall survival rates across all racial groups in a multivariate Cox regression (HR = 2.13, *p* < 0.001). Late‐stage diagnosis was also a significant predictor of increased mortality risk (HR = 3.720, *p* < 0.001). AI/ANs also had a median survival of 65 months compared to white patients who had a median survival of 90 months (*p* < 0.001).	AI/AN patients had a higher proportion of late‐stage diagnoses and significantly lower median survival compared to white patients. Multivariate analysis showed AI/ANs had over twice the risk of melanoma‐specific mortality, with late‐stage diagnosis associated with worse survival.	Income was reported on the county level, limiting the depth of this analysis on an individual level. The SEER database is also subject to underreporting and delays in reporting. Lastly, the SEER database does not have access to comorbidities or detailed treatment information.
Popp et al., 2024	Data from 2004 to 2019 were extracted from the National Cancer Database for melanoma patients. Race/ethnicity was defined as Hispanic, White, Black, Asian, Native American, or other.	After initial diagnosis, Native Americans had an average time to first treatment of 13.58 days, compared to 11.59 days for white patients. They also experienced longer times than white patients for radiation (105.65 days vs. 99.43 days), surgery (34.79 days vs 31.79 days), and chemotherapy (94.24 days vs 83.59 days). Interestingly, patients in rural areas experienced shorter times to radiation (*p* < 0.05) and chemotherapy than those in urban areas. Patients with private insurance had shortest waiting times for all treatment types except for chemotherapy, for which government insured patients had the shortest waiting time.	Native Americans faced consistently longer wait times relative to white patients across all treatment types. For chemotherapy specifically, they faced the longest time to treatment among all racial groups analyzed in this study. The findings of this study reflect the disparity in access to treatment not only for Native Americans but for Hispanic and Black patients as well.	Despite the statistical significance achieved in this study, the actual clinical impact of these findings still needs to be determined. Additionally, the NCDB is subject to missing data and inaccurate information. Lastly, the study's findings may not reflect current healthcare practices.
Zhang et al., 2024	Data from 2000 to 2019 were extracted from the SEER registries for first and second primary cutaneous melanoma patients. Race/ethnicity was defined as Hispanic, White, Black, Asian or Pacific Islander, and American Indian or Alaska Native.	Observed incidence rates for Native Americans (10.89 per 100,000, 95% CI: 10.23–11.58) were significantly higher than for Asian or Pacific Islander (1.94, 95% CI: 1.87–2.01), Black (1.35, 95% CI: 1.29–1.41), and Hispanic (7.10, 95% CI: 6.98–7.21). Relative to expected rates in the general population, they had a markedly elevated risk of second primary melanoma (standardized incidence ratio = 48.47), approximately four times that seen in white patients (standardized incidence ratio = 11.63).	These findings highlight a disproportionate burden of melanoma in the Native American population, both at initial diagnosis and in the risk of second primary melanoma.	This study is limited by surveillance bias and potential incorrect classifications of second primary melanoma.
Townsend et al., 2024	Data from 2000 to 2019 were extracted from the National Program of Cancer Registries and the SEER database for patients with cutaneous melanoma. Only non‐Hispanic American Indian/Alaska Native patients were considered.	The overall age‐adjusted incidence rate for AI/ANs was 10.7 per 100,000. 19.8% of patients were diagnosed at the regional/distant stage. Overall, the upper limb and shoulder were found to be the most common primary site (28.2%), followed by the trunk (23.2%). The upper limb and shoulder were found to be the most common primary site for men (33.5%), while the lower limb and hip were most common for women (30.2%). Incidence rates increased for AI/ANs between 40–54 years and older than 55 years with annual percent changes of 1.9 (*p* = 0.04) and 2.8 (*p* = 0.001), respectively, between 1999 and 2019.	These patterns suggest both a sex‐based difference in tumor distribution and a concerning trend of increasing melanoma burden among AI/AN populations over time. The rising incidence among patients 40 years and older underscores the need for targeted prevention and early detection efforts in this community.	This analysis did not include certain groups of AI/AN people, such as those who have not accessed care through the IHS or are not members of state‐recognized tribes. Additionally, the database is subject to potential underreporting of melanoma and does not have access to certain important clinical information, such as depth.
Fernandez et al., 2024	Data from 2004 to 2018 were extracted from the National Cancer Database for patients with primary cutaneous invasive melanoma. Only American Indian, Aleutian, or Eskimo patients were considered.	The mean age at diagnosis for AI/AN patients was 54 years. The most common primary site was the trunk (30.1%). 27.7% of patients were diagnosed in stage III (19.8%) or stage IV (7.9%). The 5 and 10‐year overall survival rates were 75.0% and 67.8%, respectively. Hazard ratios adjusted for age, sex, primary site, insurance status, CDC score, stage at diagnosis, and melanoma histology showed that no insurance (aHR = 2.9, *p* < 0.001) and Medicare (aHR = 1.86, *p* < 0.001) were significant predictors of mortality relative to private insurance. The trunk as the primary site also increased risk with an aHR of 1.72 (*p* = 0.001) and the reference as the lower extremity. Male sex also increased mortality risk (aHR = 1.37, *p* = 0.001).	This study provides insight into factors that increase the risk of mortality for AI/AN melanoma patients. It also provides data on the most common primary site and the distribution of stages at diagnosis for this population.	This study does not include melanoma‐specific survival rates. It is also impacted by missing data and potential inaccuracies within the database. Lastly, the database grouped American Indian, Aleutian, and Eskimo patients together, limiting the ability to break down analysis by individual group.
Taparra et al., 2024	Data from 2004 to 2017 were extracted from the National Cancer Database for patients between 15 and 39 years of age for the deadliest malignant neoplasms among adolescents and young adults. Race/ethnicity was defined as American Indian or Alaska Native, Asian, Black, Native Hawaiian or other Pacific Islander, and White.	AI/ANs in this cohort had the lowest proportion of patients with private insurance across all racial groups (47%) and the highest proportion that relied on Medicaid or Medicare (47%). They also faced the highest median distance to the hospital of 0.42 km. The adjusted odds ratio for late‐stage melanoma diagnosis in AI/ANs was 1.51 (95% CI: 0.77–2.79). 5‐ and 10‐year overall survival rates were 87.2% (95% CI: 79.3%–95.9%) and 87.2% (95% CI: 79.3%–95.9%), respectively, for AI/ANs and 92.9% (95% CI: 92.6%–93.2%) and 89.4% (95% CI: 89.0%–89.8%) for Whites. Hazard ratios adjusted for age, sex, income, rurality, education, year of diagnosis, distance to hospital, insurance status, Charlson‐Deyo comorbidity index, and treatment modalities showed that AI/AN patients faced increased risk of mortality compared to white patients, but this difference was not significant (aHR = 1.35, 95% CI: 0.67–2.10).	AI/AN adolescents and young adults overall face increased barriers to healthcare, such as increased distance from the hospital or insurance coverage. They also faced an increased risk of late‐stage diagnosis and mortality, specifically for melanoma, compared to white patients, but these findings were not statistically significant.	The NCDB does not have important metrics such as patient‐reported outcomes, causes of death, or psychosocial metrics. Additionally, the study did not analyze patients of Hispanic ethnicity.
Taylor et al., 2025	Data from 2000 to 2020 were extracted from the SEER database for patients with cutaneous melanoma on the head or neck. Race/ethnicity was defined as Hispanic, White, Black, Asian or Pacific Islander, and American Indian or Alaska Native.	AI/ANs in this cohort had the highest proportion of patients with an income of <$74,999 (63.7%) across all racial groups. They also had the highest proportion of rural patients (20.9%). AI/ANs had the second‐highest proportions of regional stage (15.7%), ulceration (21.1%), and Breslow thickness (54.4% with > 1.00 mm) after Black patients (28.9% regional, 28.1% ulceration, 54.5% with > 1.00 mm). White patients had 5 and 10‐year melanoma‐specific survival rates of 83.0% and 73.0%, respectively, while AI/AN patients had survival rates of 79.0% and 70.0%. Hazard ratios adjusted for age, sex, income, marital status, rural‐urban living, melanoma subtype, Breslow thickness, and disease stage showed a significantly higher risk of mortality for AI/AN patients (aHR = 1.93, *p* = 0.018).	This study demonstrates the disparity in survival rates for AI/AN patients even after controlling for income, rural residence, and disease stage. AI/ANs face a nearly two‐fold mortality risk increase relative to their white counterparts.	The SEER database does not have access to disease recurrence or insurance information. The study is also limited by the small sample sizes for minority patients.
Kim et al., 2025	Data from 2000 to 2021 were extracted from the SEER database for patients with metastatic melanoma. Race/ethnicity was defined as non‐Hispanic White, non‐Hispanic Black, non‐Hispanic Asian, non‐Hispanic American Indian/Alaska Native, and Hispanic.	The observed incidence per 100,000 for AI/ANs in 2020 and 2021 was 8.35 and 11.56, respectively, the highest among all other minority groups. There was a 20.1% decrease from the expected number of cases in 2020, likely due to missed diagnoses during the pandemic. AI/ANs had the most drastic difference in expected and observed cases in 2020 across all races. In 2021, there were 6.5% more melanoma cases than expected, which was the highest among all races as well. Time to treatment was 18 days shorter than expected for AI/ANs in 2020, but these findings may not be generalizable due to the small sample size (*n* = 14).	This study suggests that AI/ANs may have been particularly affected by low melanoma detection during the pandemic, indicated by the largest magnitude decrease in observed compared to expected cases. Other findings specific to AI/ANs are limited since survival and mortality were not analyzed by race.	This study had a limited follow‐up time, particularly for metrics such as survival and mortality. Survival time was limited to only one year, restricting the ability to compare this study's results alongside other literature.

### 4.1 Meta‐Analysis

**Figure 2:**
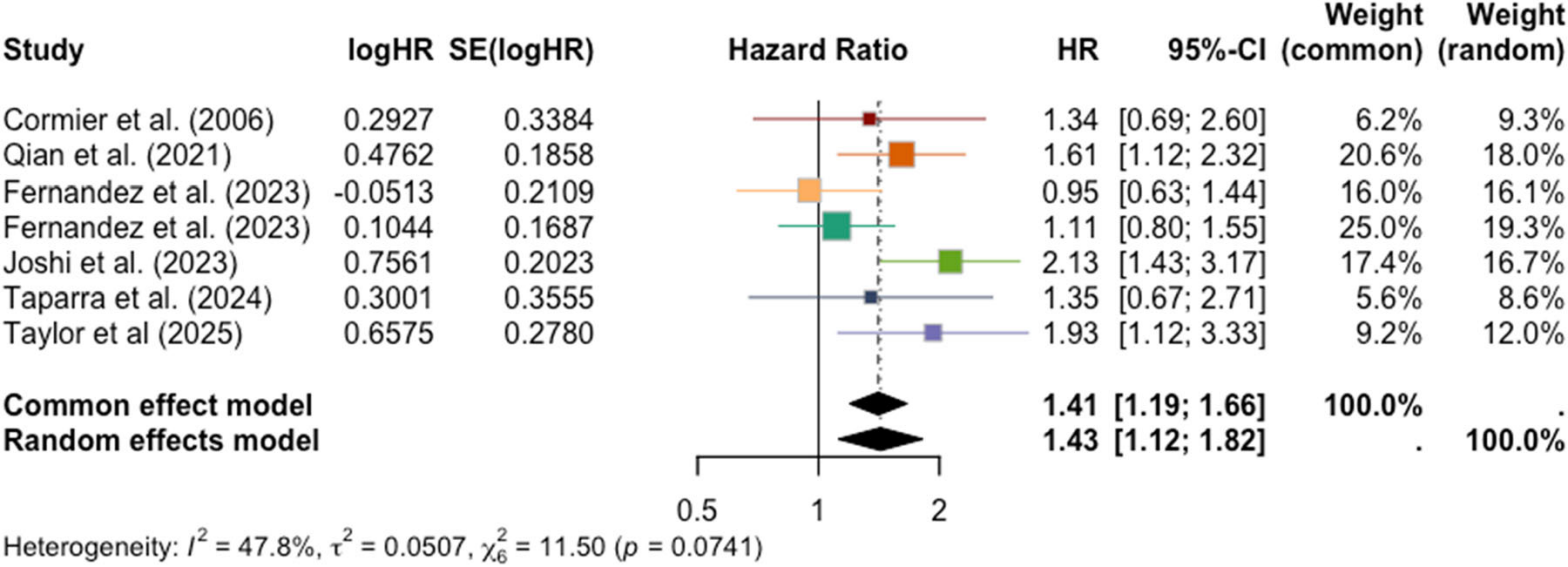
Forest plot displaying adjusted hazard ratios (aHRs) and 95% confidence intervals for melanoma among AI/AN patients compared to white patients.

**Figure 3:**
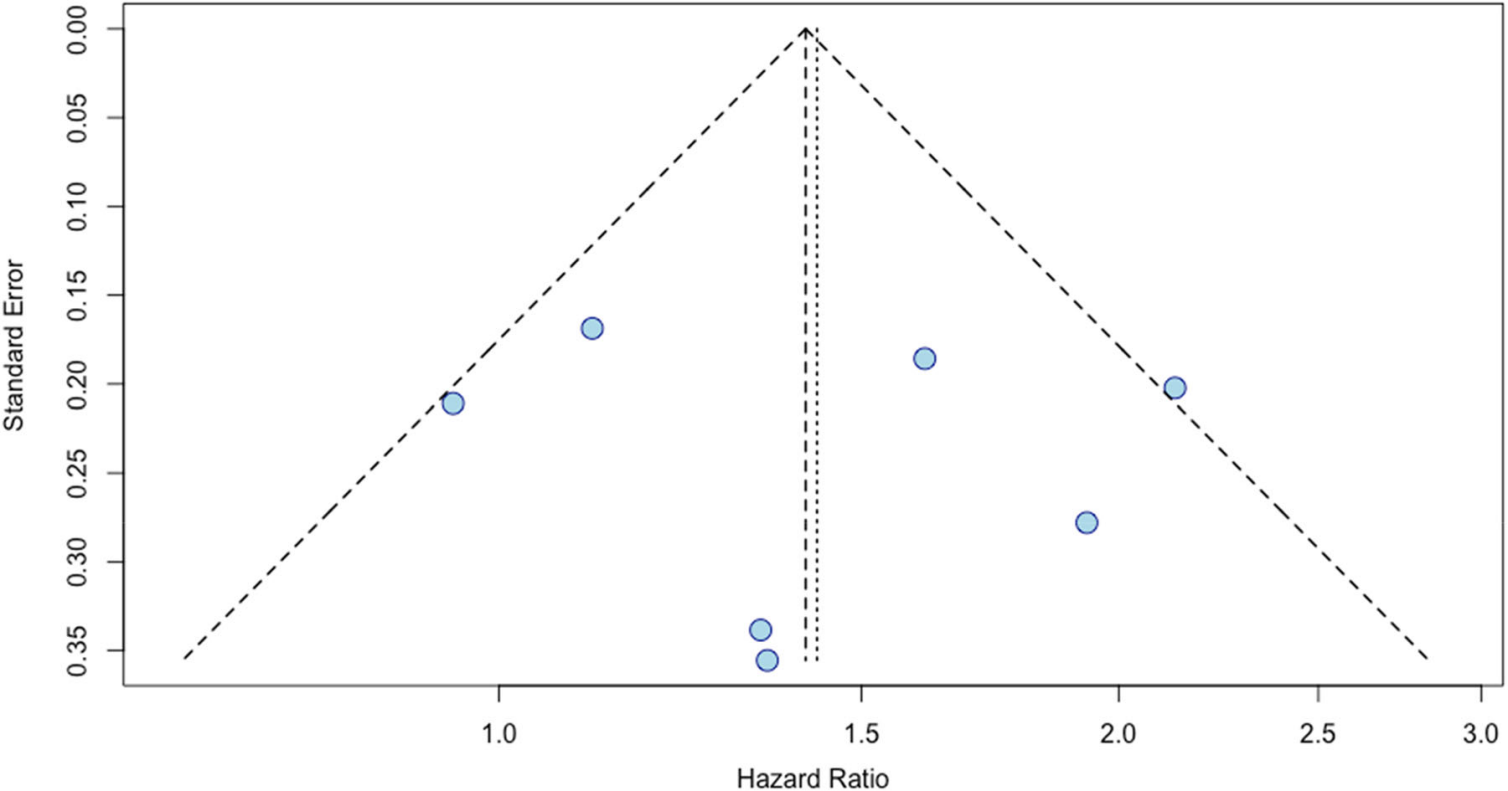
Funnel plot assessing potential publication bias in studies reporting adjusted hazard ratios (aHRs) for melanoma in AI/AN patients compared to white patients.

The meta‐analysis conducted on adjusted hazard ratios across seven studies (Cormier et al., 2006; Qian et al., 2021; Fernandez et al., 2023a; Fernandez et al., 2023b; Joshi et al., 2023; Taparra et al., 2024; Taylor et al., 2025) demonstrates statistically significant disparities in melanoma survival [8, 9, 10, 11, 12, 13, 14]. The combined aHR from the random effects model was 1.43 (95% CI: 1.12–1.82; *p* = 0.0041), revealing the elevated mortality risk that AI/AN patients face relative to their white counterparts (Figure 2). There was moderate heterogeneity found across studies (I^2^ = 47.8%). All studies except Fernandez et al. (2023a) consistently demonstrated this increased risk, but only three were able to find statistical significance [10]. There was no significant publication bias, as indicated by the funnel plot (Figure 3). The findings of this meta‐analysis highlight the need to better understand the causes of these disparities and to develop targeted approaches that enhance healthcare access and promote equity for AI/AN communities.

**Figure 4:**
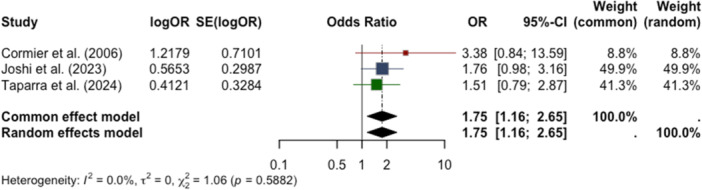
Forest plot displaying adjusted odds ratios (aORs) and 95% confidence intervals for late‐stage melanoma diagnosis in AI/AN patients relative to white patients.

**Figure 5:**
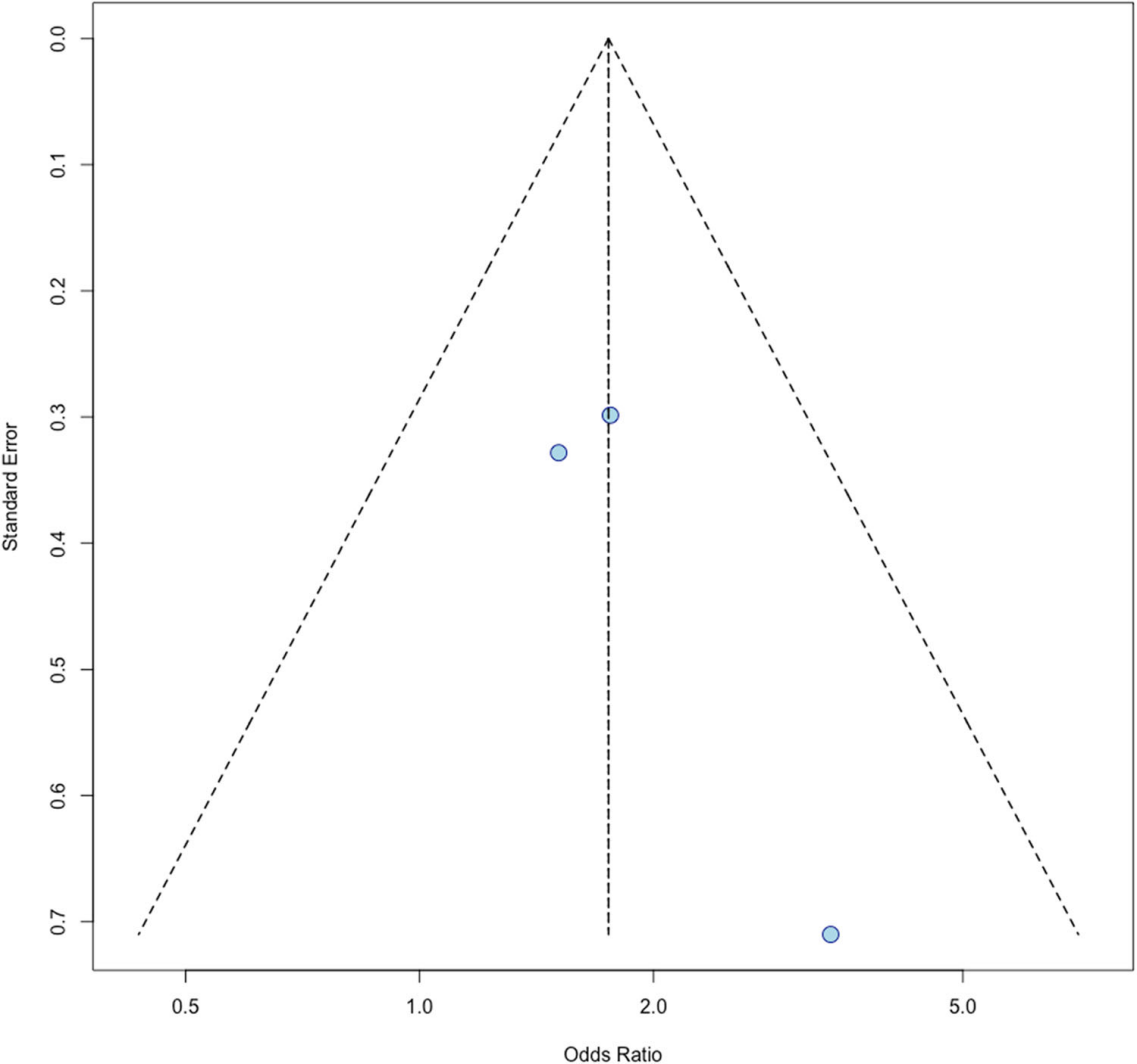
Funnel plot assessing potential publication bias in studies reporting adjusted odds ratios (aORs) for late‐stage melanoma diagnosis among AI/AN patients.

The second meta‐analysis of adjusted odds ratios from three studies (Cormier et al., 2006; Joshi et al., 2023; Taparra et al., 2024) found that AI/AN patients had a significantly higher chance of late‐stage diagnosis relative to white patients [8, 12, 13]. The random effects model produced a combined OR of 1.75 (95% CI: 1.16–2.65; *p* = 0.0080) (Figure 4). There was no heterogeneity found across studies (I^2^ = 0%). This observed increase in risk of late‐stage diagnosis is consistent among the reported findings, but none of the individual studies reached statistical significance. There was limited publication bias, as indicated by the funnel plot (Figure 5)

**Figure 6:**
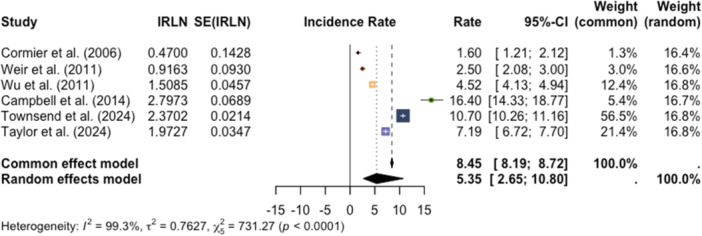
Forest plot displaying annual age‐adjusted incidence rates of melanoma among AI/AN patients across included studies with corresponding confidence intervals.

**Figure 7:**
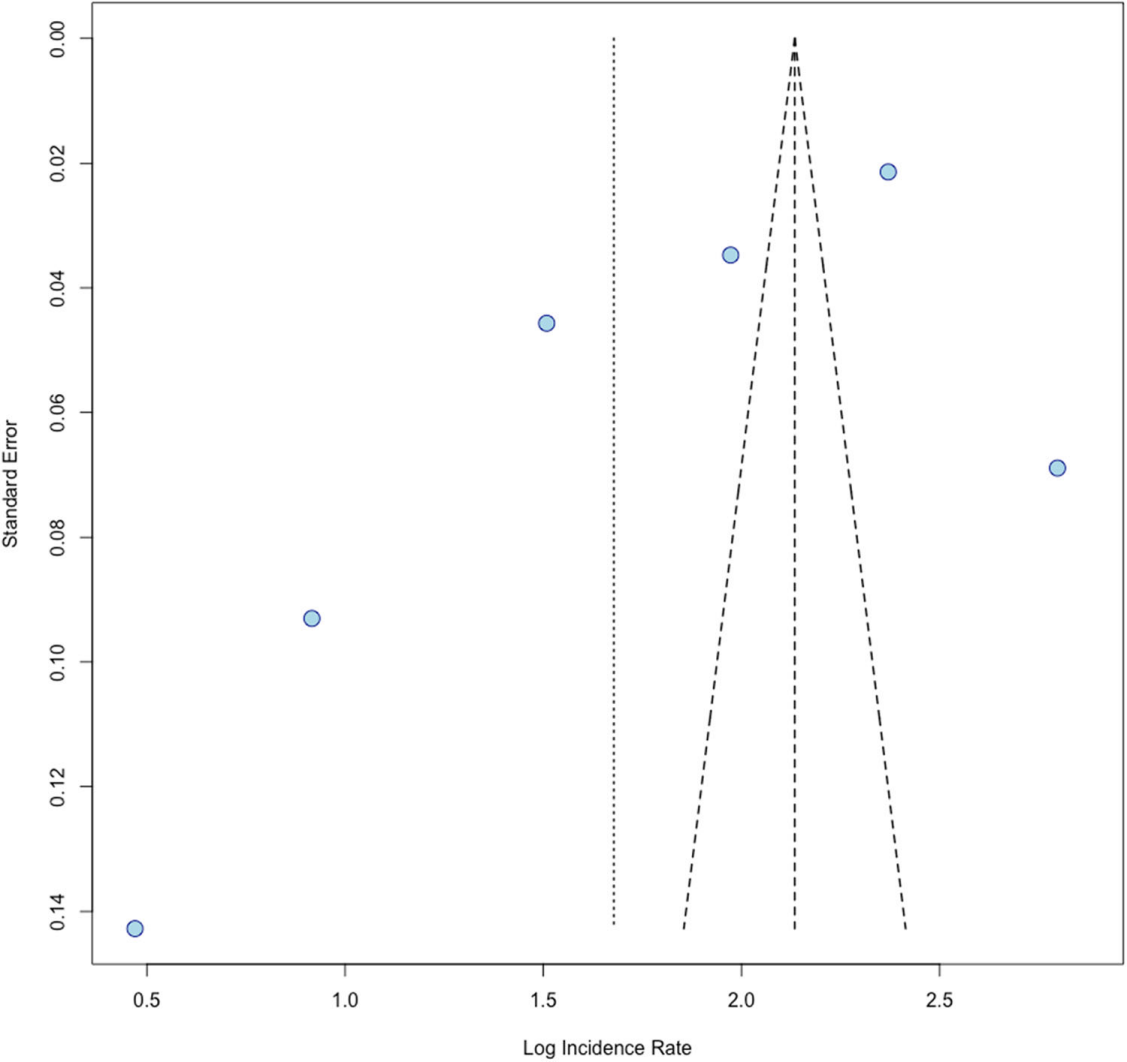
Funnel plot assessing variability and potential asymmetry in reported annual age‐adjusted incidence rates of melanoma among AI/AN patients.

The final meta‐analysis was conducted to aggregate annual age‐adjusted incidence rates for melanoma in AI/AN populations. The random effects model applied across six studies (Cormier et al., 2006; Weir et al., 2011; Wu et al., 2011; Campbell et al., 2014; Townsend et al., 2024; Taylor et al., 2025) found an incidence rate of 5.35 per 100,000 (95% CI: 2.65–10.80) (Figure 6) [8, 14, 15, 16, 17, 18]. There was high heterogeneity across studies (I^2^ = 99.3%), and the funnel plot indicated publication bias (Figure 7). The observed heterogeneity demonstrates the need for improved accuracy for melanoma reporting within this patient population.

#### 4.1.1 Disparities in Melanoma at Diagnosis

Studies consistently report worse prognostic factors for American Indian and Alaska Native (AI/AN) patients, particularly with respect to stage at diagnosis, Breslow thickness, and the presence of skin ulceration. These disparities are evident across multiple large‐scale analyses. Cormier et al. (2006) conducted a retrospective cross‐sectional analysis of the Surveillance, Epidemiology, and End Results (SEER) database, including 49,772 cutaneous melanoma cases, 52 of whom were AI/AN [8]. The study found that AI/AN individuals were 3.38 times more likely to present with stage IV disease (95% CI: 0.84–13.59).

Similarly, Wu et al. (2011) examined data from 567 AI/AN patients among a total of 277,235 cutaneous melanoma cases in the SEER and National Program of Cancer Registries (NPCR) registries. They found that a significantly higher proportion of AI/AN patients were diagnosed at regional or distant stages (23.9% vs. 12.1%), and tumors exceeding 4.0 mm in thickness were also more common in this group (11.2% vs. 4.6%) [16].

Expanding on this trend, Campbell et al. (2014) analyzed data from the Oklahoma Central Cancer Registry (OCCR), examining 4,681 patients, including 214 AI/ANs across two time periods [17]. A higher proportion of AI/AN patients were diagnosed at regional or distant stages in both periods: 27.5% versus 20.6% from 1997 to 2001, and 20.0% versus 14.0% from 2005 to 2009 (*p* = 0.030).

Baldwin et al. (2016) studied 6,874 patients from the OCCR, 341 of whom were AI/AN [19]. They found that 20.1% of AI/AN patients were diagnosed at the regional or distant stage compared to 15.1% of white non‐Hispanic patients (*p* = 0.02).

In a national study, Qian et al. (2021) analyzed data from 398,034 patients in the SEER database, including 809 non‐Hispanic AI/AN (NHAIAN) individuals [9]. They found that 18.6% of NHAIANs were diagnosed at regional or distant stages, compared to 12.6% of non‐Hispanic whites. NHAIANs also had thicker tumors (mean Breslow thickness of 1.49 mm vs. 1.22 mm) and more frequent skin ulceration (16.2% vs. 12.8%).

Adding further evidence, Fernandez et al. (2023b) analyzed data of 205,125 male melanoma patients, 201 of whom were AI/AN, from the National Cancer Database [11]. In their study, the median Breslow thickness among AI/AN patients was 1.15 mm (IQR: 0.42–3.06), compared to 0.88 mm (IQR: 0.40–2.00) for white patients. A larger proportion of AI/AN individuals were diagnosed at stage III or IV (29.1%) compared to white patients (21.1%). Additionally, AI/ANs had higher rates of positive lymph node status (19.4% vs. 13.8%) and skin ulceration (24.7% vs. 20.6%).

More recently, Joshi et al. (2023) reported that 16.5% of AI/AN patients were diagnosed at stage III or IV compared to 10.4% of white patients, although this difference did not reach statistical significance [12]. Among 37,984 total patients analyzed, 115 were AI/AN. In a multivariate logistic regression, the odds ratio for late‐stage diagnosis among AI/AN patients was 1.76 (*p* = 0.060).

Taparra et al. (2024) analyzed the National Cancer Database, focusing on 36,724 patients aged 15–39, of whom 66 were AI/AN [13]. The study reported an adjusted odds ratio of 1.51 (95% CI: 0.77–2.79) for melanoma diagnosis at stage III or IV in AI/AN patients.

Similarly, Townsend et al. (2024) analyzed data from 2,151 AI/AN individuals from the NPCR and SEER [18]. They found that 19.8% of these patients were diagnosed at regional or distant stages. Fernandez et al. (2024) examined data from 429 AI/AN patients in the National Cancer Database and found that 27.7% were diagnosed at stage III (19.8%) or stage IV (7.9%) [20].

Finally, Taylor et al. (2025) analyzed cutaneous melanoma of the head and neck using SEER data, which included 58,936 patients and 115 AI/AN individuals [14]. Compared to whites, AI/AN patients had higher rates of regional stage disease (15.7% vs. 10.7%), ulceration (21.1% vs. 15.6%), and thicker tumors (54.4% vs. 37.7% with Breslow thickness > 1.00 mm).

#### 4.1.2 Disparities in Melanoma Outcomes

Disparities in stage at diagnosis among AI/AN patients also translate into differences in survival outcomes. Several studies have documented consistently lower survival rates and higher mortality risks in this population. Cormier et al. (2006) were among the first to highlight this issue [8]. Their study found that AI/AN patients had significantly lower 5‐year overall and melanoma‐specific survival rates, 69.8% and 81.0%, respectively, compared to 79.3% and 89.6% for white patients (*p* < 0.001). Although aHRs accounting for demographic and clinical variables indicated a higher risk of melanoma‐specific mortality for AI/AN patients (aHR = 1.34), this difference did not reach statistical significance (95% CI: 0.69–2.6).

Expanding the analysis to mortality patterns, Bristow et al. (2013) examined 155,571 U.S. death certificates, including 234 from AI/AN individuals [21]. They observed that AI/AN patients died at a younger average age (62.5 years) compared to white patients (66.1 years). Moreover, the age‐adjusted melanoma mortality rate for AI/ANs was the highest among all nonwhite racial groups at 0.88 per 100,000.

Qian et al. (2021) provided a longitudinal perspective by examining changes in survival disparities over time [9]. Their analysis of SEER data revealed increasing mortality risk for non‐Hispanic AI/ANs (NHAIANs) compared to whites across three time periods. The adjusted hazard ratios were 1.12 (1975–2000), 1.15 (2000–2009), and 1.61 (2010–2016), with the latter showing a statistically significant difference (95% CI: 1.12–2.32). Notably, in the 2010–2016 period, NHAIANs had elevated mortality risks at localized (aHR = 2.14) and regional disease (aHR = 2.86) relative to non‐Hispanic white patients. These categories were defined by the SEER summary staging systems, where localized disease is associated with no spread of the tumor and regional disease indicates spread to nearby lymph nodes.

Supporting these findings, Joshi et al. (2023) identified AI/AN patients as having the highest risk of poor overall survival across all racial groups in a multivariate Cox regression (aHR = 2.13, *p* < 0.001) [12]. The median survival for AI/AN patients diagnosed at stages III‐IVc was 65 months, which was significantly lower than the 90‐month median survival observed among white patients diagnosed at the same stages (*p* < 0.001).

In contrast to these trends, Fernandez et al. (2023a) analyzed data from 163,315 patients in the National Cancer Database, including 163 AI/AN individuals [10]. They found no significant difference in mortality between non‐Hispanic AI/AN women and white women, with an adjusted hazard ratio of 0.95 (*p* = 0.823). This finding suggests that sex‐specific or subpopulation‐specific factors may influence outcomes.

Additional analysis showed that survival disparities also extended to male patients [11]. After adjusting for age, race, ethnicity, primary tumor site, insurance status, Charlson‐Deyo comorbidity score, stage at diagnosis, melanoma histology, ulceration, surgery, systemic therapy, and radiation, AI/AN males had an aHR of 1.11 compared to white males (*p* = 0.541). Five‐year overall survival rates were also lower among AI/AN males (68.5%) relative to white males (75.1%).

Further insight into survival disparities was provided by Taparra et al. (2024), who studied a younger cohort of patients aged 15–39 [13]. They found that 5‐ and 10‐year overall survival rates for AI/AN patients were both 87.2%, compared to 92.9% and 89.4% for white patients. Although AI/AN patients faced an elevated mortality risk (aHR = 1.35), the difference was not statistically significant (95% CI: 0.67–2.10) after adjusting for a wide range of sociodemographic and clinical factors. Fernandez et al. (2024) also reported lower 5‐ and 10‐year overall survival rates among AI/AN patients, 75.0% and 67.8%, respectively [20].

In contrast to analyses of national datasets, Rosenthal et al. (2023) examined a cohort of 14,614 insured melanoma patients in California [22]. Among the 27 Native American patients included, only one melanoma‐specific death was reported. The adjusted hazard ratio for mortality among Native Americans with insurance outside of Kaiser Permanente was 1.84 (95% CI: 0.26–13.25). The overall aHR for insured Native Americans in the registry was 0.98 (95% CI: 0.14–7.00). The HRs reported by Rosenthal et al. were not included in the present study's meta‐analysis due to the cohort excluding uninsured patients.

Lastly, Taylor et al. (2025) analyzed melanoma‐specific survival using SEER data and found that AI/AN patients had 5‐ and 10‐year survival rates of 79.0% and 70.0%, respectively, compared to 83.0% and 73.0% among white patients [14]. After adjusting for age, sex, income, rurality, marital status, melanoma subtype, Breslow thickness, and disease stage, AI/AN patients had significantly higher mortality risk (aHR = 1.93, *p* = 0.018).

#### 4.1.3 Incidence Rates

The meta‐analysis found an age‐adjusted incidence rate of 5.35 per 100,000 for AI/ANs [8, 14, 15, 16, 17, 18]. Melkonian et al. (2022) analyzed trends from the CDC and SEER databases for non‐Hispanic AI/AN and non‐Hispanic white patients [23]. The authors found that from 1999 to 2017, the incidence rate increased by 53% among white females and 186% among AI/AN females, and by 54% among white males and 98% among AI/AN males. Similarly, Townsend et al. (2024) found that incidence rates increased for AI/ANs between 40 and 54 years and older than 55 years with annual percent changes of 1.9 (*p* = 0.04) and 2.8 (*p* = 0.001), respectively [18].

Two studies reported observed incidence rates, which were not included in the meta‐analysis. Zhang et al. (2024) assessed 542,929 patients from the SEER program for first and second primary cutaneous melanoma, with 1094 AI/ANs [24]. The authors reported that observed incidence rates for Native Americans (10.89 per 100,000, 95% CI: 10.23–11.58) were significantly higher than for Asian or Pacific Islander (1.94, 95% CI: 1.87–2.01), Black (1.35, 95% CI: 1.29–1.41), and Hispanic (7.10, 95% CI: 6.98–7.21) patients. Relative to expected rates in the general population, they had a four times higher risk of second primary melanoma compared to white patients (standardized incidence ratio = 48.47 vs. 11.63). Kim et al. (2025) assessed the incidence of melanoma in 673,681 patients from the SEER program. Across both years, 438 AI/ANs were included in the analysis [25]. The authors reported observed incidence rates for AI/ANs in 2020 and 2021 were 8.35 and 11.56 per 100,000, respectively.

#### 4.1.4 Barriers to Care

Beyond differences in diagnosis and survival outcomes, several studies underscore disparities in access to care, insurance coverage, treatment delays, and socioeconomic factors among AI/AN melanoma patients. Insurance disparities have been consistently documented across multiple datasets. Campbell et al. (2014) reported that 40.7% of AI/AN patients were covered by Medicare [17]. Medicaid coverage was more common among AI/ANs than white patients (7.8% vs. 4.1%, *p* < 0.001), while more white patients relied on private insurance (31.1% vs. 24.7%, *p* < 0.001). Similarly, Fernandez et al. (2023b) found that among male melanoma patients, 43.4% of AI/ANs had Medicare, and another 43.4% had private insurance [11]. However, 7.2% of AI/ANs relied on Medicaid (vs. 2.6% of whites), and 6.1% were uninsured.

Taparra et al. (2024) further highlighted these disparities, showing that AI/AN patients had the lowest rate of private insurance (47%) and the highest reliance on Medicaid or Medicare (47%) compared to all other racial groups [13]. Differences in insurance status directly translated into differences in survival, as shown by Fernandez et al. (2024) [20]. The authors reported that patients without insurance had nearly triple the mortality risk of those with private insurance (aHR = 2.9, *p* < 0.001), while Medicare coverage was associated with a 1.86‐fold increase in mortality risk (*p* < 0.001).

Popp et al. (2024) examined timeliness of treatment and found that after diagnosis, AI/AN patients experienced longer delays than white patients across all major treatment types [26]. On average, they waited 13.58 days to begin treatment, compared to 11.59 days for white patients. Delays extended to radiation (105.65 vs. 99.43 days), surgery (34.79 vs. 31.79 days), and chemotherapy (94.24 vs. 83.59 days), though the clinical significance of these delays remains unclear. Notably, rural patients began radiation and chemotherapy sooner than those in urban areas. Patients with private insurance generally experienced the shortest wait time, except for chemotherapy, where government‐insured patients had faster access.

Treatment disparities may also be influenced by geographic barriers. Taparra et al. (2024) found that AI/ANs faced the longest median distance to the hospital of 0.42 km [13]. Fernandez et al. (2023b) similarly reported that AI/ANs lived the furthest away from their diagnosing institution (24.5 miles; 95% CI: 10.7–72.2), a factor that could contribute to delayed care or treatment interruptions [11].

Socioeconomic differences compound these challenges. AI/ANs had lower median incomes than Hispanic, Asian, and white patients, with only 23% of AI/ANs earning more than $63,000 a year [11]. Similarly, Taylor et al. (2025) revealed that 63.7% of AI/AN patients earned less than $74,999 annually, the highest proportion across all racial groups [14]. Additionally, 20.9% lived in rural areas, highlighting geographic and economic vulnerabilities that can affect access to dermatologic services and timely cancer care.

Disparities in utilization of diagnostic procedures have also been reported. Martinez et al. (2012) conducted a multivariate logistic regression using SEER data from 8,775 patients and found that Native American patients had the lowest likelihood of undergoing sentinel lymph node biopsy (SLNB) compared to other racial groups (OR = 0.19, *p* = 0.019) [27]. However, this analysis was based on a small AI/AN sample size (*n* = 9), which may limit the robustness of the findings.

Even after diagnosis, disparities persist in long‐term surveillance. Qian et al. (2021) showed that AI/AN patients had a shorter median follow‐up period of 74 months compared to 86 months for white patients, suggesting possible gaps in posttreatment monitoring and survivorship care [9].

Lastly, results from Kim et al.'s study demonstrate that the COVID‐19 pandemic could have further exacerbated disparities [25]. The authors reported a 20.1% decrease in observed melanoma cases among AI/AN patients in 2020, the largest drop across all racial groups. Consequently, AI/AN patients saw the highest rebound in melanoma diagnoses in 2021, with cases rising 6.5% above expected levels. This temporal pattern suggests a backlog of delayed or missed screenings that disproportionately affected AI/ANs throughout the pandemic.

## 5 Discussion

The results of this systematic review and meta‐analyses demonstrate significant disparities in prognostic factors and mortality risk for AI/ANs diagnosed with cutaneous melanoma. Despite improvements in melanoma‐specific survival rates in recent years, AI/AN patients remain at significantly elevated risk for both mortality (combined aHR = 1.43) and late‐stage diagnosis (combined adjusted OR = 1.75) [8, 9, 10, 11, 12, 13, 14]. The temporal trend showing these disparities is worsening rather than improving: mortality risk for AI/ANs increased from aHR = 1.12 (1975–2000) to 1.61 (2010–2016) [9].

Our review found that a higher proportion of AI/ANs were also found to present with skin ulceration (aHR = 1.77, *p* < 0.001) and higher Breslow thickness (median HR = 0.23 for T1 vs T4, *p* < 0.05), factors that have both been shown to increase risk of melanoma‐related mortality [10, 11, 28]. The increasing incidence of melanoma in AI/AN patients is alarming, with incidence rates rising 186% among AI/AN females compared to 53% among white females, and 98% among AI/AN males versus 54% among white males from 1999 to 2017 [23]. Several studies reported the second‐highest incidence rates for AI/ANs after white patients [15, 24, 25].

The combination of rising incidence rates with our newly established evidence of significantly delayed diagnosis creates a concerning trajectory for AI/AN melanoma outcomes, underscoring the urgent need for population‐specific interventions targeting early detection. Many AI/AN individuals receive care through the Indian Health Service (IHS), tribally operated health programs, or Urban Indian Organizations (UIOs) [29, 30]. Tribal health centers are central to care delivery in many AI/AN communities. While UIOs serve substantial urban Native populations, they receive only 1% of the IHS budget [30].

These underfunded systems frequently lack advanced dermatologic capabilities, forcing reliance on the Purchased and Referred Care (PRC) program, which prioritizes conditions by medical urgency and may delay skin cancer evaluations [31]. These structural challenges place AI/AN patients at a systemic disadvantage when it comes to early melanoma detection and prompt intervention.

Geographic barriers further compound these systemic issues. AI/AN patients face the greatest average distance to hospitals or diagnosing institutions compared to other racial groups [11, 13]. This isolation not only delays initial evaluations of lesions but also reduces the likelihood of routine skin cancer screening, particularly when compounded by limited access to private transportation or public transit. These geographic challenges translate into measurable treatment delays: AI/AN patients waited an average of 13.58 days to begin treatment compared to 11.59 days for white patients, with delays extending across other treatment modalities including surgery (34.79 vs 31.79 days), radiation (105.65 vs 99.43 days), and chemotherapy (94.24 vs 83.59 days) [26].

In addition to physical separation from medical institutions, Guadagnolo et al. have shown that Native American cancer patients more generally experience less satisfaction and more mistrust than their white counterparts (*p* = 0.0001) [32]. This lack of confidence extends to researchers, as well, potentially contributing to the lack of documentation of health outcomes for AI/ANs [33]. The history of displacement and cultural disruption that Native Americans have faced contributes to a reluctance to trust medical institutions and the scientific community as a whole. This hesitancy to undergo preventive healthcare procedures such as screenings or annual check‐ups may contribute to the worsened prognostic factors observed within AI/AN melanoma patients.

For patients who do reach a healthcare provider, timely access to dermatologic care remains a challenge. Tsang et al. (2006) reported long wait times for dermatology appointments across the U.S., even for patients presenting with melanoma concerns [34]. This national shortage of dermatologists disproportionately impacts rural and underserved populations, where access is already strained. The combination of these factors increases the likelihood of diagnosis at more advanced stages of disease, which has been shown to elevate mortality risk by up to ten times (aHR at stage IV = 10.26, *p* < 0.001) [10].

Insurance disparities represent another critical barrier to equitable care. Taparra et al. (2024) found that AI/AN patients have the lowest proportion of private insurance among all racial groups (47%) [13]. Several studies reported significant portions of AI/ANs who relied on Medicare or Medicaid, ranging from 47% to 50.6% [11, 13, 17]. Additionally, 6.1% of AI/AN patients were uninsured, compared to only 2.4% for white patients [11].

While Medicare and Medicaid provide critical coverage for many individuals, they also have more constraints on provider networks and reimbursement rates. These limitations can result in longer wait times for treatment and reduced access to specialists. Martinez et al. (2012) demonstrated concrete results of this, with AI/ANs facing significantly lower odds of receiving sentinel lymph node biopsy (OR = 0.19) [27].

Additionally, uninsured AI/AN patients face nearly triple the mortality risk of those with private insurance (aHR = 2.9, *p* < 0.001), while those on Medicare face an 86% higher risk of mortality compared to privately insured patients (aHR = 1.86, *p* < 0.001) [20]. This difference highlights the role of insurance type not just as a socioeconomic indicator but as an independent predictor of survival.

The findings from Rosenthal et al. (2023) provide important context for interpreting survival disparities in AI/AN patients [22]. Their study, which was restricted to insured adults with melanoma in California, suggests that insurance may be a key driver of previously observed racial disparities. The lack of mortality differences in Rosenthal's integrated health system cohort suggests that enhanced access to comprehensive care frameworks may help narrow outcome disparities. This finding underscores how insurance adequacy and system integration could address many observed inequities.

The shorter median follow‐up duration observed among AI/AN patients (74 months vs. 86 months) for white patients raises significant concerns about continuity of care and survivorship support in this population [9]. AI/AN patients may be at greater risk for recurrence or delayed detection of secondary malignancies.

More recently, the disruption caused by the COVID‐19 pandemic reveals how fragile and uneven access to timely cancer diagnosis can be during times of crisis. Kim et al. (2025) found that AI/AN patients experienced the most pronounced drop in diagnoses during 2020 and the steepest rebound in 2021 [25]. This rebound effect likely reflects a catch‐up period for missed or delayed screenings, demonstrating how the pandemic intensified pre‐existing vulnerabilities within this patient population. Future emergency planning must create more resilient and accessible screening systems to prevent disproportionate care disruptions among marginalized communities.

The findings of this systematic review and meta‐analysis highlight the longstanding structural limitations in the healthcare systems that serve AI/ANs. Efforts to reduce racial disparities in melanoma outcomes must prioritize early detection interventions alongside policy‐level reforms to improve insurance equity. The statistical confirmation of delayed diagnosis provides a clear target for intervention: screening programs, provider education, and community outreach initiatives designed to promote earlier melanoma detection in AI/AN populations now have evidence‐based justification for implementation and funding. Improving reimbursement structures and enhancing care coordination for publicly insured patients has the potential to lessen the disparity in melanoma‐related outcomes between AI/AN and white patients. Additional interventions, such as telehealth services and increased funding for tribal health infrastructure, are also critical to expand access to care. Given the worsening temporal trends for incidence rates and the compounding effects of recent pandemic disruptions, urgent action is needed to prevent further widening of these disparities. Without such measures, AI/AN communities will continue to face disproportionate barriers to timely, effective melanoma care.

The present study represents the first meta‐analysis to demonstrate statistically significant evidence that AI/AN patients face higher likelihood of late‐stage melanoma diagnosis compared to white patients (aOR = 1.75, *p* = 0.0080). While individual studies consistently reported this trend across multiple datasets, none achieved statistical significance independently due to limited AI/AN sample sizes. By aggregating data across multiple high‐quality studies, our meta‐analysis overcame these power limitations and transformed what was previously considered suggestive evidence into definitive proof of this critical disparity.

The clinical relevance of our findings is strengthened by the adjustment for important confounding variables. The studies in our meta‐analyses provided adjusted hazard ratios and odds ratios that controlled for demographic factors, clinical characteristics, and socioeconomic variables, allowing for more precise estimates of the independent effect of AI/AN ethnicity on melanoma outcomes. This adjustment for confounders increases confidence that observed disparities reflect true differences in outcomes

Finally, our study's focus on AI/AN populations addresses a significant gap in the current literature. AI/AN patients represent a relatively small but important population that has been understudied in melanoma research, partly due to the challenges of achieving adequate sample sizes. By demonstrating the value of meta‐analytic approaches for studying health disparities in smaller population groups, our work provides a framework that can be applied to investigate disparities in other underrepresented populations and cancer types.

However, this review is subject to several limitations. Our meta‐analysis of incidence rates and adjusted odds ratios of late‐stage diagnosis show high (I^2^ = 99.3%) and moderate (I^2^ = 47.8%) heterogeneity, respectively. The observed variability among studies may limit the interpretability of these two meta‐analyses. Due to limited research in this field, older studies such as Cormier et al., 2006 were included in the meta‐analyses, which could affect the generalizability of the results [8]. Additionally, several studies had small sample sizes of AI/ANs with melanoma, ranging from as low as nine patients. Lastly, all studies included were retrospective in nature, relying on databases that may have inaccuracies or incomplete information.

Future studies should evaluate melanoma rates across racial groups prospectively, limiting potential race and ethnicity misclassifications and ensuring more complete data. Further research is also needed to determine the most effective paths to lessening the disparity in melanoma outcomes within AI/AN communities.

## 6 Conclusions

This systematic review and meta‐analysis illuminate the significant disparity that AI/ANs diagnosed with cutaneous melanoma face in terms of prognostic factors and mortality risk. Despite improvements in AI/AN survival rates and advances in melanoma treatment, disparities persist. The findings of this meta‐analysis provide the necessary evidence to justify targeted funding for AI/AN melanoma screening programs and enhanced dermatologic services within tribal health systems. Without such measures, AI/AN communities will continue to face disproportionately poor melanoma outcomes.

## Synopsis

This systematic review and meta?analysis highlight the disparity in survival outcomes and stage at diagnosis for American Indian and Alaska Native melanoma patients in the United States. The results of 20 observational studies were examined thematically and quantitatively in this comprehensive analysis.

## Data Availability

The data that support the findings of this study are available in Google at https://www.google.com/. These data were derived from the following resources available in the public domain: ‐ Embase, https://www.embase.com/landing?status=yellow ‐ PubMed, https://pubmed.ncbi.nlm.nih.gov/ ‐ Scopus, https://www.scopus.com/home.uri.
